# Recent advances in the understanding of male lower urinary tract symptoms (LUTS)

**DOI:** 10.12688/f1000research.8638.1

**Published:** 2016-04-21

**Authors:** Arman A. Kahokehr, Peter J. Gilling

**Affiliations:** 1 Department of Urology, Wellington Hospital, Wellington, New Zealand; 2 Head of School, Bay of Plenty Clinical School, Tauranga, New Zealand

**Keywords:** male lower urinary tract symptoms, LUTS, detrusor underactivity, nocturia

## Abstract

In this review, we have looked at three important areas in understanding male lower urinary tract symptoms. These are improvement in terminology, detrusor underactivity, and nocturia. Benign prostatic hyperplasia leading to bladder outlet obstruction has been covered in a previous review.

## Introduction

Lower urinary tract symptoms (LUTS) are a progressive and age-related, but not sex- or organ-specific, group of ‘complaints’ and comprise a combination of storage, voiding, and post-micturition symptoms ^1^. LUTS are highly prevalent in the population ^2^. They cause bother and impair quality of life ^2, 3^. They are strongly associated with ageing and represent a major health burden ^3^.

The natural history of LUTS is dynamic. In some individuals, LUTS will persist and worsen over time and for others will wax, wane, and remit. In this review, we look at three important areas in the understanding of LUTS often not covered in the traditional descriptions. These are improvements in the terminology and diagnostics of LUTS, detrusor underactivity (DU), and nocturia.

The management of benign prostatic hyperplasia (BPH) leading to benign prostatic enlargement (BPE) and subsequent bladder outlet obstruction (BOO) has been covered in a previous F1000 review ^4^.

Although LUTS in men are traditionally thought to relate to BOO secondary to BPH, studies have shown that LUTS are often unrelated to prostate disease ^1^. Bladder dysfunction may also cause LUTS, including detrusor overactivity and DU, as well as other structural or functional abnormalities of the urinary tract ^5^. In addition, many non-urological conditions contribute to LUTS, especially nocturia ^1^.

## Improvements in terminology

Clinical symptoms of BPH have been described as ‘prostatism’. This terminology was sufficient, but perhaps oversimplified, for another era ^6^. However, not all men with histological BPH would develop clinical sequelae. Prostatism ** (incorrectly) implies an organ-specific source of symptoms. Recent advances in the understanding of these symptoms have concentrated on getting the terminology internationally accepted and standardised. The term LUTS describes the patients’ symptoms without implying a cause and has replaced older terms such as ‘prostatism’ ^7^.

Male LUTS have traditionally been ascribed to BOO, which often is caused by BPE resulting from BPH. The transition in terminology with an emphasis on symptoms-based terminology greatly aids the clinical mindset and the understanding of the differences between clinical and laboratory-based diagnostics and helps dispel the misconceptions that all LUTS in men are caused by BPE.

## Detrusor underactivity

DU is defined by the International Continence Society (ICS) as a voiding contraction of reduced strength or duration (or both), which prolongs urination or prevents complete emptying of the bladder within a ‘normal’ period of time (or both) ^8^. DU is associated with voiding LUTS and a high post-void residual, which may predispose to urinary tract infection and acute urinary retention. The true prevalence is likely under-recognised, and the aetiology is not well understood. Several factors are likely to play a part, and there are several current pathophysiological models affecting myogenic function and neural control mechanisms as well as the efferent and afferent innervation.

### Ageing model

It is currently unknown whether ageing is the primary cause or a condition necessary for the development of DU. The association between DU and ageing is well established, but there are conflicting data on bladder function and morphology in ageing animals. Afferent nerve density declines in ageing animals ^9^; however, the age-related increase in urothelial transmitter release within the human bladder ^10^ has not been reproduced in animal preparations. In both rats and mice, contractility either is diminished or increases with age ^11, 12^. Detrusor muscle loss usually ^12^, but not always, increases with age.

### Altered afferent sensory and efferent neuronal model

Impaired bladder contractility has been traditionally regarded as a major aetiological factor behind DU. However, over time, the bladder has decreased bladder afferent innervation, which is associated with DU and hence suggests complex pathology ^13^. The urothelium, detrusor muscle, interstitial cells, and ganglia form a mechanical sensor and transducer system which activates afferent nerve fibres ^13^. Each of these components could have an impact on lower urinary tract function by altering the release of neurotransmitters, thereby altering the excitability of sensory fibres and the contractility of the detrusor muscle in the urinary bladder. These afferent inputs monitor bladder volume and determine detrusor contraction during the voiding phase. By ending prematurely, these afferent signals may prematurely terminate the voiding reflex (as seen in diabetic cystopathy). Detrusor contraction force and duration are a result of efferent nerve activity, which in turn is dependent on sensory input, hence the potential for impaired afferent function to cause DU.

### Ischaemia/oxidative stress models

*In vitro* as well as *in vivo* animal studies show a correlation between oxidative stress and impaired contractility. Atherosclerosis-induced chronic bladder ischaemia significantly reduces detrusor contractility in animals ^14– 16^. It is still unknown whether these models will lead to therapeutic targets in humans.

### Obstruction and bladder over-distension

BOO has traditionally been seen as a prerequisite to LUTS in the ageing male population. However, whether a patient develops a higher post-void residual or eventual urinary retention is not dependent only on the grade of BOO. Animal studies have shown the relationship between bladder tissue mass and altered contractile responses to BOO ^17, 18^. Initially, there is muscle hypertrophy and hyperplasia leading to a thick-walled bladder, resulting in decreased tissue oxygen tension and chronic ischaemia. Contractility increases to compensate; however, after a variable period, detrusor function is impaired and results in a decompensating phase.

Deterioration of bladder function proceeds slowly, and the reversibility of function after removal of the obstruction is often not seen once a patient is in the decompensated state. This may mean that reversing obstruction may not reverse the detrusor contractility that is lost.

### Neurogenic models

Incomplete emptying is common in patients with bladder dysfunction caused by neurological disease such as multiple sclerosis, Parkinson’s disease, and multiple system atrophy ^19, 20^. Dysfunction of the central control mechanism and voiding reflex may lead to DU by affecting the perception, integration, and outflow. In these models, DU can span a spectrum from a slightly decreased ability to generate pressure to a bladder that cannot generate any pressure. Though useful for understanding specific scenarios, these models are unlikely to be applied to a wider non-neurological model for patients with DU.

### Diagnosis

Urodynamic tests are used to diagnose DU, either by assessing the relationship between bladder pressure and urinary flow or by interrupting voiding to measure detrusor pressure changes in isovolumetric conditions. Diagnostic criteria are based on urodynamic measurements relating to bladder contractility such as maximum flow rate and detrusor pressure at maximum flow ( Table 1). Other estimates rely on mathematical formulas to calculate isovolumetric contractility indices or urodynamic ‘stop tests’. Most methods have practical disadvantages or are poorly validated. Contraction strength is only one aspect of bladder voiding function, however. The others are the speed and persistence of the contraction, which have not yet been incorporated in a widely accepted international diagnostic regime.

**Table 1.** Diagnostic urodynamic criteria used to define detrusor underactivity.

First author, year	Diagnostic criteria	Prevalence of detrusor underactivity ^a^
Nitti *et al.* ^28^, 2002	Bladder outlet obstruction index <20 and Q _max_ <12 ml/s	9%
Kaplan *et al.* ^29^, 1996	P _det_@Q _max_ <45 cm H2O and Q _max_ <12 ml	23% (5% acontractile)
Abarbanel and Marcus ^30^, 2007	P _det_@Q _max_ <30 cm H2O and Q _max_ <10 ml	48% (male) 12% (female)
Jeong *et al.* ^31^, 2012	Bladder contractility index <100 (men) Q _max_ <12, P _det_@Q _max_ <10 (women)	40% (male) 13% (female)
Fusco *et al.* ^32^, 2001	P _det_@Q _max_ <30 and Q _max_ <12	10%

^a^Percentage with an acontractile detrusor. P _det_@Q _max_, detrusor pressure at the time of maximum flow; Q _max_, maximum flow.

Treatments for DU have poor efficacy and tolerability and often fail to improve quality of life; muscarinic receptor agonists, in particular, have limited efficacy and frequent adverse effects. Bladder emptying might be achieved through Valsalva straining and intermittent or indwelling catheterization. Novel stem cell-based therapies have been attempted; however, new drugs that increase contractility are currently largely conceptual, and the complex pathophysiology of DU, the difficulty of achieving organ specificity of treatment, the limited availability of animal models, and the subjective nature of current outcome measures must be addressed as part of the development of such agents.

## Nocturia

In normal adult physiology, the amount of urine produced at night is less than the functional bladder capacity, hence the ability to sleep at night without having to wake to void. This is based on adequate anti-diuretic hormone (ADH) production. Our knowledge of the pathophysiology of nocturia has not dramatically changed recently; however, it is recognised that nocturia is increasingly complex and multifactorial in aetiology. These factors can be divided into (a) bladder storage problem, (b) nocturnal polyuria, (c) global polyuria, and (d) mixed disorder or sleep disorder or both. In addition to the known causes of LUTS, several recent advances may help shed light on this very common and bothersome symptom. Nocturia is increasingly important and independently associated with sleep-disordered breathing ^21^.

### Circadian defects in the secretion of anti-diuretic hormone

Compared with normal controls, patients with nocturia have little or no diurnal variation in urine output and have greater nocturnal urine production. This is associated with the lack of a nocturnal increase in ADH level ^22^. The exact physiological reasons underlying this defect in ADH secretion have not been fully elucidated. When used carefully, intranasal desmopressin may improve nocturnal polyuria and can extend the time to first void (an important aspect concerning sleep quality) ^23^.

### Neurogenic or non-neurogenic detrusor overactivity

Overactive bladder (OAB) syndrome is defined by the ICS as symptoms of urinary urgency, with or without urge incontinence, usually with increased daytime frequency and nocturia. Urgency is thought to be the primary driver of the syndrome of OAB. Nocturnal cystometrograms show the relationship with nocturnal detrusor overactivity and nocturia ^24^. Urgency also increases the risk of having nocturia by 5- to 7-fold ^25^; however, most patients with nocturia do not report urgency. These data point to a mixed and complex pathophysiology, which is not fully understood.

### Associations with metabolic syndrome

Obesity is associated with a two to threefold increased risk for nocturia ^26, 27^, and patients with nocturia have a higher risk of diabetes ^25^. The association between diabetes/obesity and sleep apnoea is well established, but the association between nocturia and hypertension and coronary artery disease is less well elucidated. However, the increase in the prevalence of metabolic syndrome is likely to lead to further interest in the association between nocturia and this global problem.

## Summary

The understanding of LUTS is evolving and becoming increasingly complex. Consensus group reports point out that LUTS increase with age and are prevalent in both male and female patients ^1^. LUTS are neither gender nor organ specific and are sometimes age related and sometimes progressive. There is a need to further investigate and understand LUTS, its causes, the resulting morbidity, and the therapeutic strategies necessary for this very common problem.

## Abbreviations

ADH, anti-diuretic hormone; BOO, bladder outlet obstruction; BPE, benign prostatic enlargement; BPH, benign prostatic hyperplasia; DU, detrusor underactivity; ICS, International Continence Society; LUTS, lower urinary tract symptoms; OAB, overactive bladder.
